# A Bias‐Tunable Multispectral Photodetector Based on a GaN/Te*_x_* Se_1‐_ *_x_* Homo‐Type Heterojunction with a Unidirectional Barrier

**DOI:** 10.1002/advs.202417428

**Published:** 2025-03-07

**Authors:** Weijie Liu, Meng Peng, Maohua Chen, Yongming Zhao, Yiye Yu, Pengcheng Jian, Zunyu Liu, Yuhui Zeng, Yuang Luo, Xiantai Tian, Zhiwei Gao, Jiangnan Dai, Changqing Chen, Feng Wu, Weida Hu

**Affiliations:** ^1^ Wuhan National Laboratory for Optoelectronics Huazhong University of Science and Technology Wuhan 430074 China; ^2^ State Key Laboratory of Infrared Science and Technology Shanghai Institute of Technical Physics Chinese Academy of Sciences Shanghai 200083 China; ^3^ School of Optical and Electronic Information Huazhong University of Science and Technology Wuhan 430074 China

**Keywords:** bias‐tunable photoresponse, gallium nitride, multispectral photodetector, tellurium‐selenium alloys

## Abstract

Multispectral detection technology captures characteristic spectral information across various wavebands, exhibiting substantial application potential. However, most currently reported multispectral photodetectors rely on intricate dual‐ or multi‐junction structures, severely limiting material thickness, doping concentration, and band alignment design, thereby impeding widespread adoption. In this study, a bias‐tunable multispectral photodetector featuring a straightforward single‐junction design is introduced. The device comprises a Te*_x_* Se_1‐_ *_x_*/GaN homo‐type heterojunction with a unidirectional barrier. This structure effectively suppresses the majority‐carrier dark current, yielding a low reverse dark current of ≈10^−12^ A and a high rectification ratio of up to 10^5^. By adjusting the bias polarity and magnitude, the spectral response range of the device can be broadened from ultraviolet (UV) to short‐wave infrared. Notably, the photodetection performance is exceptional: at 0 V bias, the device exhibits a responsivity of 0.25 A W^−1^ and a specific detectivity of 5.04 × 10^11^ cm Hz^1/2^ W^−1^ under 365 nm illumination; at −2 V bias, it achieves a responsivity of 0.58 A W^−1^ and a specific detectivity of 2.64 × 10^9^ cm Hz^1/2^ W^−1^ under 1060 nm illumination. Leveraging the bias‐tunable spectral response characteristic of the device, proof‐of‐concept imaging is successfully demonstrated. This research presents a simplified and economical method for fabricating multispectral photodetectors.

## Graphical abstract

A bias‐tunable multispectral photodetector based on a Te*_x_* Se_1‐_ *_x_*/GaN homo‐type heterojunction with a unidirectional barrier is proposed. By modulating the bias polarity and magnitude, the device's spectral response range can be extended from UV to the SWIR region. The proof‐of‐concept imaging demonstrates the device's excellent target recognition capability in complex application scenarios.

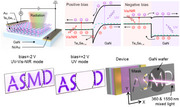

## 1 Introduction

Optoelectronic detection technology, which utilizes optical signals for information perception, represents a cutting‐edge advancement that has significantly propelled the rapid development of modern information society. However, as application scenarios become increasingly complex and demands continue to rise, photodetectors with fixed spectral response ranges have begun to reveal shortcomings in practical applications, such as low interference resistance, high false alarm rates, and poor environmental adaptability.^[^ ^1^, ^2^, ^3^ ^]^ To enhance the anti‐jamming capabilities of detection systems and to reflect a richer set of intrinsic information about the targets in order to improve target recognition accuracy and efficiency, multispectral detection technology has begun to develop progressively. In particular, its advantages in all‐weather and full‐time operation have demonstrated broader application value in areas such as autonomous vehicles,^[^ ^4^ ^]^ medical diagnoses,^[^ ^5^ ^]^ satellite reconnaissance,^[^ ^6^ ^]^ astronomical observation,^[^ ^7^ ^]^ etc. Traditional multispectral detection technology typically requires the integration of photodetectors operating at different spectral bands within a single system to work in collaboration. However, this scheme requires a substantial number of additional optical components such as lenses, prisms, and gratings to separate the wavelength components, which greatly increases the size, complexity, and instability of the system.^[^ ^8^, ^9^, ^10^ ^]^ Consequently, the development of miniaturized monolithic integrated multispectral photodetectors by heterogeneously integrating two or more kinds of materials sensitive to a different spectral band has gradually become a focal point of international research.^[^ ^11^, ^12^, ^13^, ^14^, ^15^ ^]^

The earliest bias‐tunable multispectral photodetectors can be traced back to the late 20th and early 21st centuries, during which researchers achieved multispectral infrared detection by stacking III–V‐based quantum wells or superlattices with different response wavelengths.^[^ ^16^, ^17^, ^18^, ^19^ ^]^ In 2008, D. Hofstetter et al. successfully integrated AlGaN‐based ultraviolet (UV) detectors and GaN/AlN superlattice infrared detectors on the same substrate, enabling voltage‐tunable detection in both ultraviolet and infrared (IR) wavebands.^[^ ^20^ ^]^ However, the aforementioned multispectral photodetectors based on traditional semiconductor materials exhibit significant limitations, such as their complex structure, high material growth costs, and cryogenic operation requirements. Furthermore, constrained by the selection rules of electronic transitions, these devices possess low quantum efficiency and are unable to absorb vertical incident radiation.

Subsequently, the rise of low‐dimensional materials and the advent of the nanotechnology era have provided a broader range of material options and diverse device structures for optoelectronic device design.^[^ ^21^, ^22^, ^23^, ^24^, ^25^, ^26^ ^]^ In recent years, significant research progress has been made in the field of multispectral detection. Notable advancements include HgTe colloidal quantum dots (CQDs) back‐to‐back photodiodes for dual‐band infrared imaging,^[^ ^27^ ^]^ miniaturized computational spectrometer with an electrically tunable van der Waals (vdW) junction,^[^ ^28^ ^]^ hybrid structure dual‐band infrared detectors combining PbS CQDs with BP/MoS₂ heterojunction,^[^ ^29^ ^]^ spectra‐adapted vision sensors based on arrays of back‐to‐back photodiodes,^[^ ^3^ ^]^ and 2D/3D vdW heterostructure based multispectral photodetectors,^[^ ^30^, ^31^, ^32^ ^]^ among others. However, most of the reported multispectral photodetectors are based on complex dual‐junction or multi‐junction structures, imposing severe restrictions on the selection of material thickness and doping concentration, as well as the design of band alignments. This significantly hinders the widespread applicability of the multispectral photodetectors. In addition, current research primarily focuses on IR and visible‐infrared (Vis‐IR) multispectral detection, with few reports on ultraviolet–visible–infrared (UV–Vis–IR) multispectral photodetectors. This scarcity is primarily due to energy band matching constraints, which make it challenging to realize high responsivity in both ultraviolet and infrared wavebands simultaneously.^[^ ^33^, ^34^, ^35^ ^]^ These issues pose substantial challenges in the fabrication of UV–Vis–IR multispectral photodetectors.

Recently, tellurium (Te) as an emerging single‐element semiconductor has stuck out from numerous narrow‐bandgap infrared detection materials.^[^ ^36^, ^37^, ^38^ ^]^ One of the advantages is its unique structure, which is composed of one‐dimensional helical molecular chains bound by vdW forces.^[^ ^39^, ^40^ ^]^ This implies the absence of surface dangling bonds, thereby circumventing the issue of lattice matching in traditional heteroepitaxial growth. Furthermore, by forming tellurium‐selenium alloys (Te*_x_* Se_1‐_ *_x_*) with variable Se composition, the bandgap of Te can be continuously tuned from 0.35 to 1.9 eV,^[^ ^41^, ^42^ ^]^ corresponding to the response cutoff wavelength ranging from mid‐wave infrared to visible light. Additionally, Te*_x_* Se_1‐_ *_x_* exhibits low melting points and high saturated vapor pressures, enabling the production of high‐quality thin films through simple thermal evaporation techniques.^[^ ^43^, ^44^, ^45^ ^]^ These characteristics make Te*_x_* Se_1‐_ *_x_* one of the best candidate materials for hetero‐integrated photodetectors.

In this work, we report a bias‐tunable multispectral photodetector that operates at room temperature with merely a simple single‐junction structure. With a delicate design of band alignment, a Te*_x_* Se_1‐_ *_x_*/GaN homo‐type heterojunction with a unidirectional barrier is constructed. In this structure, the majority‐carrier dark current is effectively suppressed by the unidirectional barrier, resulting in a low reverse dark current of ≈10^−12^ A and a high rectification ratio up to ≈10^5^. By adjusting the bias polarity and magnitude, the direction of carrier transport and carrier collection efficiency can be regulated, thereby switching the device from UV detection mode to UV–Vis–IR broadband detection mode. Moreover, the designed device demonstrates a competitive detection performance. At 0 V bias, the device exhibits a responsivity of 0.25 A W^−1^ and a specific detectivity of 5.04 × 10^11^ cm Hz^1/2^ W^−1^ under 365 nm illumination. At a bias of −2 V, a responsivity of 0.58 A W^−1^ and a specific detectivity of 2.64 × 10^9^ cm Hz^1/2^ W^−1^ are achieved under 1060 nm illumination. The device working principle is further analyzed by technology computer‐aided design (TCAD) simulations. Based on these results, we demonstrate the great potential application of the device in target recognition in complex scenarios and deep‐sea detection technology. In summary, this work paves a new way for the design of novel multispectral photodetectors.

## 2 Results and Discussion

### 2.1 Device Design and Structure Characterization

**Figure 1:**
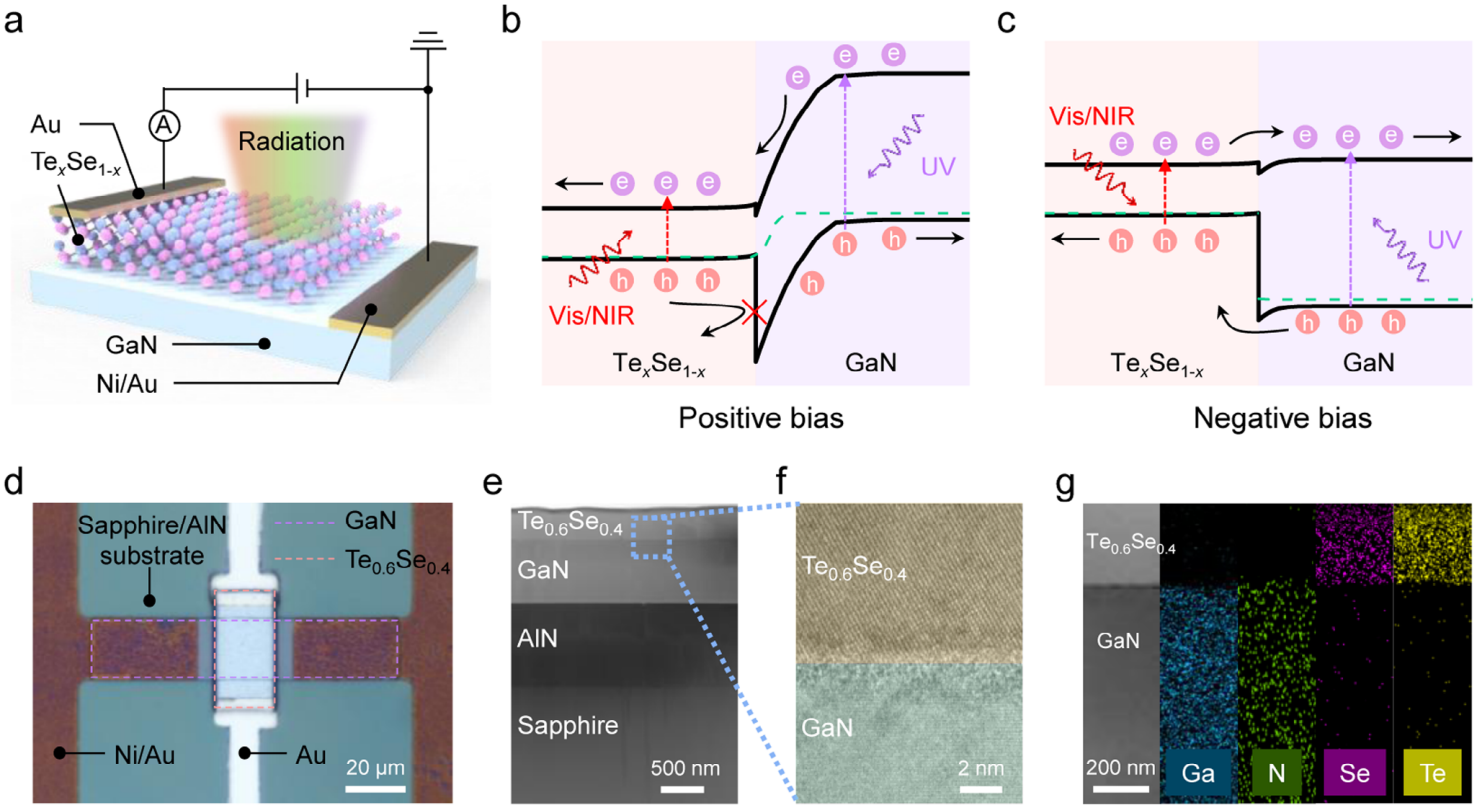
Design of the bias‐tunable multispectral photodetector. a) The device structure of the bias‐tunable multispectral photodetector. b,c) Energy band diagrams for the device under positive and negative bias, respectively. d) Optical microscope image of the device. The contours of GaN and Te*_x_* Se_1‐_ *_x_* are highlighted by green and orange dashed lines, respectively. e) Cross‐sectional TEM image of the device. f) High‐resolution cross‐sectional TEM image of the Te*_x_* Se_1‐_ *_x_*/GaN interface. g) The EDS mapping of the Te*_x_* Se_1‐_ *_x_*/GaN heterojunction.

**Figure** **1**a illustrates the bias‐tunable multispectral photodetector we designed, consisting of a Te*_x_* Se_1‐_ *_x_*/GaN p‐P homo‐type heterojunction. The detailed fabrication process of the device can be found in the Experimental Section and Figure S1 (Supporting Information). The bandgaps and work functions of Te*_x_* Se_1‐_ *_x_* and GaN can be calculated from the absorption spectrum and the ultraviolet photoelectron spectroscopy (UPS), respectively (Figures S2 and S3, Supporting Information). According to these results, the band alignments of Te*_x_* Se_1‐_ *_x_* and GaN before and after contact are deduced, as shown in Figure S4 (Supporting Information). When Te*_x_* Se_1‐_ *_x_* and GaN are brought in physical contact, the minority electrons in Te*_x_* Se_1‐_ *_x_* are injected into GaN while the majority holes in GaN move into Te*_x_* Se_1‐_ *_x_* until an equilibrium state is reached. As a result, a hole accumulation region is formed at Te*_x_* Se_1‐_ *_x_* side whereas a wide depletion region is formed at the GaN side.^[^ ^46^, ^47^ ^]^ Meanwhile, a large valence‐band offset of this heterojunction forms a unidirectional hole barrier, resulting in the blockage of hole transport from Te*_x_* Se_1‐_ *_x_* to GaN.^[^ ^48^, ^49^ ^]^ While, due to the relatively small conduction‐band offset, the electrons can flow almost unimpeded in both directions. Utilizing the unidirectional blocking effect of the valence band barrier, we can modulate the spectral response range of the device by merely switching the bias polarity. The working principle of bias‐tunable multispectral detection in this device is further elucidated through the energy band diagrams under positive and negative bias. Here we specify that the voltage direction of positive bias is from Te*_x_* Se_1‐_ *_x_* to GaN, and the opposite for negative bias. Under positive bias, the photogenerated carriers in GaN excited by UV radiation can be collected by the external circuit to form a photocurrent (Figure 1b). However, the photogenerated holes in Te*_x_* Se_1‐_ *_x_* are impeded by the barrier and ultimately recombine with an equal number of electrons from the external circuit. That is to say, in this case, the device cannot respond to the radiation absorbed by the Te*_x_* Se_1‐_ *_x_*, and only sense to the UV radiation absorbed by GaN. Therefore, the spectral response range of the device contains only the UV region under positive bias. While the opposite occurs under negative bias (Figure 1c). Since the direction of the applied electric field for negative bias is opposite to that of the built‐in electric field, a large enough negative bias needs to be applied to reverse the transport direction of the photogenerated carriers. Thus, the electron‐hole pairs generated in both Te*_x_* Se_1‐_ *_x_* and GaN can be collected unimpeded without the blockage of a barrier, resulting in a UV–Vis–IR broadband response in this device.

Figure 1d shows the optical microscope image of the designed device. The crystal quality and elemental composition of Te*_x_* Se_1‐_ *_x_* and GaN were further uncovered by Raman spectra and X‐ray photoelectron spectroscopy (XPS), respectively (Figures S5 and S6, Supporting Information). The Te atomic ratio in the Te*_x_* Se_1‐_ *_x_* sample was determined to be *x* ∼ 0.57 from the XPS results. The X‐ray diffraction (XRD) patterns of the Te*_x_* Se_1‐_ *_x_* thin film exhibit three distinct characteristic peaks, with the most intense peak corresponding to the (100) crystal plane, indicating the film's high crystalline quality (Figure S7, Supporting Information). To verify the high quality of the heterostructure, a cross‐sectional transmission electron microscopy (TEM) measurement was conducted (Figure 1e,f). The High‐resolution TEM image displays that the interface between GaN and Te*_x_* Se_1‐_ *_x_* is clean and free of distinct mixing or diffusion layers. Additionally, energy‐dispersive x‐ray spectrometry (EDS) mapping of Te*_x_* Se_1‐_ *_x_*/GaN interface was captured and shown in Figure 1g, where components of Ga, N, Te, and Se are clearly identified with uniform distribution on both sides, demonstrating sharp and clean heterojunction interface. The corresponding selected area electron diffraction (SAED) pattern of the Te*_x_* Se_1‐_ *_x_* thin film shows a few sets of diffraction spots (Figure S8, Supporting Information), further indicating its polycrystalline structure.

### 2.2 Optoelectronic Performance Characterization

**Figure 2:**
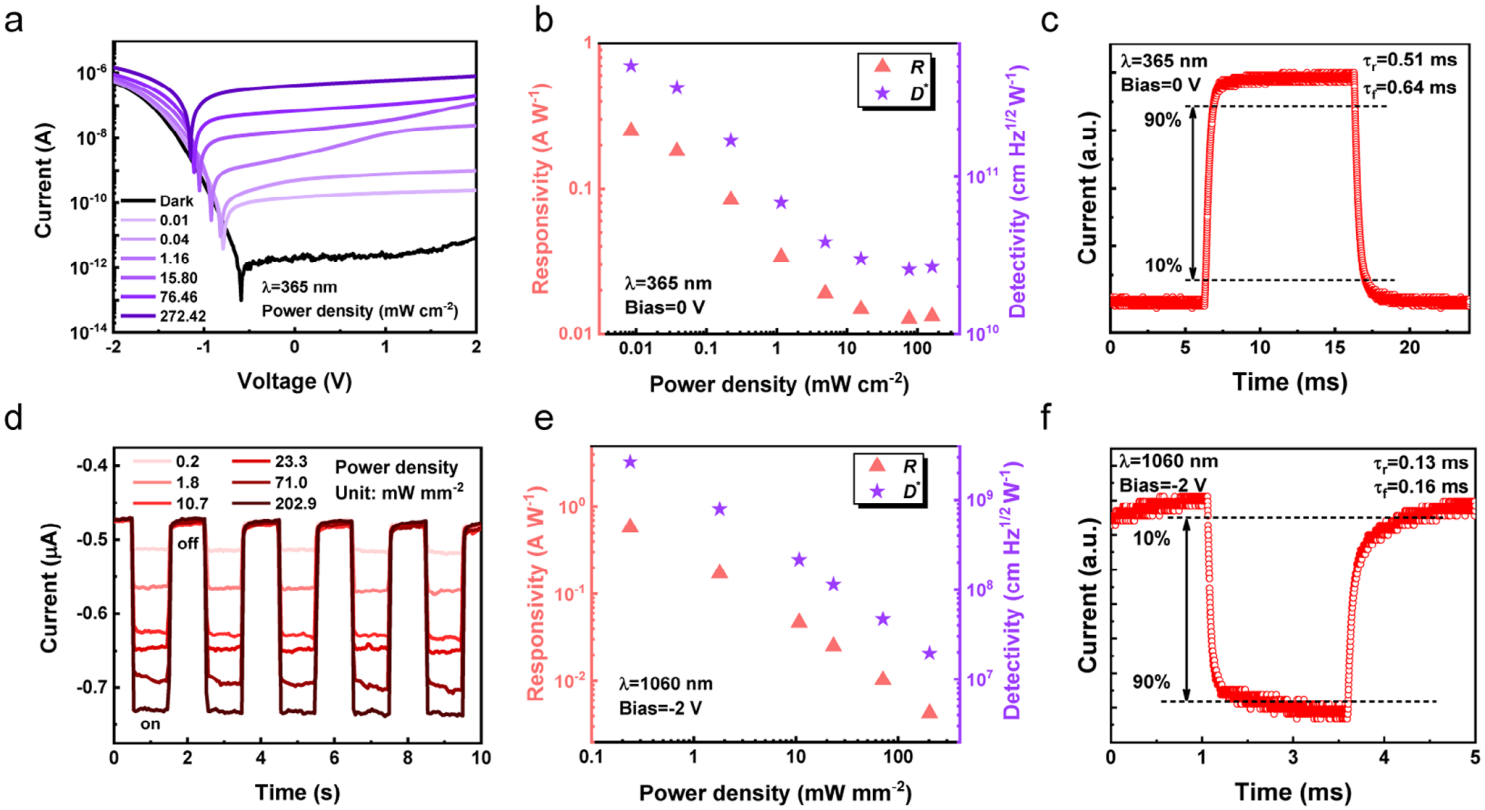
Optoelectronic Performance of Te*_x_* Se_1‐_ *_x_*/GaN p‐P Heterojunction. a) The *I*–*V* curve of the Te*_x_* Se_1‐_ *_x_*/GaN p‐P heterojunction under 365 nm illumination with increasing power densities. b) *R* and *D* ^*^ at zero bias versus power densities of 365 nm wavelength. c) rise and fall times measured at zero bias with 365 nm illumination. d) Time‐dependent photoresponse of the Te*_x_* Se_1‐_ *_x_*/GaN p‐P heterojunction at −2 V bias with a different power density of 1060 nm laser. e) *R* and *D* ^*^ versus power densities of 1060 nm wavelength. f) rise and fall times measured at −2 V bias with 1060 nm laser illumination.

Ohmic contacts are essential for the fabrication of high‐performance photovoltaic detectors.^[^ ^26^, ^50^, ^51^ ^]^ In order to obtain low‐resistance ohmic contacts of p‐GaN, we tested the current–voltage relation (*I‐* *V*) curves of p‐GaN after thermal annealing at different temperatures and proved that ohmic contacts were formed when the annealing temperature was 700 °C by calculating the specific contact resistivity (Figure S9, Supporting Information). The *I*‐*V* curves of p‐GaN and Te*_x_* Se_1‐_ *_x_* forming the heterojunction were provided in Figure S10 (Supporting Information), indicating the realization of ohmic contacts for both materials. On the basis of the high absorption of GaN and Te*_x_* Se_1‐_ *_x_* in the UV and IR wavebands, respectively, the optoelectronic performance of the device was then characterized. The classic *I*–*V* characteristics of the device under different powers of a 365 nm UV illumination are shown in **Figure** **2**a. It can be observed that the device exhibits a backward‐rectifying behavior and negative open‐circuit voltages, which is consistent with the optoelectronic characteristics of homo‐type heterojunctions.^[^ ^46^, ^47^ ^]^ Moreover, attributed to the suppression of majority‐carrier dark current by the valence band barrier,^[^ ^48^, ^49^ ^]^ an extremely low dark current of ≈10^−12^ A and high photo‐to‐dark‐current ratio up to ≈10^6^ were achieved under positive bias. In contrast, Te*_x_* Se_1‐_ *_x_*/GaN p‐N heterojunctions suffer from large reverse leakage currents, resulting in a poor rectification characteristic (Figure S11, Supporting Information). This is reasonable since for Te*_x_* Se_1‐_ *_x_*/GaN p‐N heterojunctions, the unidirectional barrier at the valence band loses its blocking effect to the majority holes in Te*_x_* Se_1‐_ *_x_* under negative bias, as shown in Figure S12 (Supporting Information).

To quantify the UV detection performance of the Te*_x_* Se_1‐_ *_x_*/GaN p‐P heterojunction device, we calculated the responsivity (*R*), and specific detectivity (*D* ^*^) of the device under zero bias by the following formulas:

(1) $$R=\frac{I_{ph}}{PS}$$

(2) $$D^{*}=\frac{\sqrt{Sf}}{NEP}$$

where *I*_ph_ denotes the photocurrent, P is the laser power density, S is the effective area of the device, *f* is bandwidth and *NEP* is noise equivalent power. The *NEP* represents the minimum light power that can be detected from the total noise by a photodetector. It is generally expressed as *NEP* = *i*_n_/*R*, where *i*_n_ is the noise power density. The relevant noise power density spectra of the Te*_x_* Se_1‐_ *_x_*/GaN p‐P heterojunction are given in Figure S13 (Supporting Information). Figure 2b displays the variation of *R* and *D* ^*^ of the device with light power density. The maximum *R* and *D* ^*^ values of 0.25 A W^−1^ and 5.04 × 10^11^ cm Hz^1/2^ W^−1^ were obtained under the light power density is 8 µW cm^−2^, respectively. The response time is another critical performance indicator of a photodetector, which can be divided into rise time (τ_r_) and fall time (τ_f_). The τ_r_ and τ_f_ are respectively defined as the duration when the photocurrent increases from 10% to 90% of the peak value and drops from 90% to 10% of the peak value. Accordingly, under 365 nm UV illumination, the device exhibits a τ_r_ of 0.51 ms and τ_f_ of 0.64 ms, respectively (Figure 2c). Next, to estimate the NIR detection performance, we conduct the time‐dependent photoresponse test with different power densities of 1060 nm laser under −2 V bias (Figure 2d). The maximum *R* and *D* ^*^ for 1060 nm laser are 0.58 A W^−1^ and 2.64 × 10^8^ cm Hz^1/2^ W^−1^, respectively (Figure 2e). Figure 2f shows that the device exhibits a fast response speed for 1060 nm laser with τ_r_ of 0.16 ms and τ_f_ of 0.13 ms, respectively.

### 2.3 Bias‐Tunable Multispectral Detection

**Figure 3:**
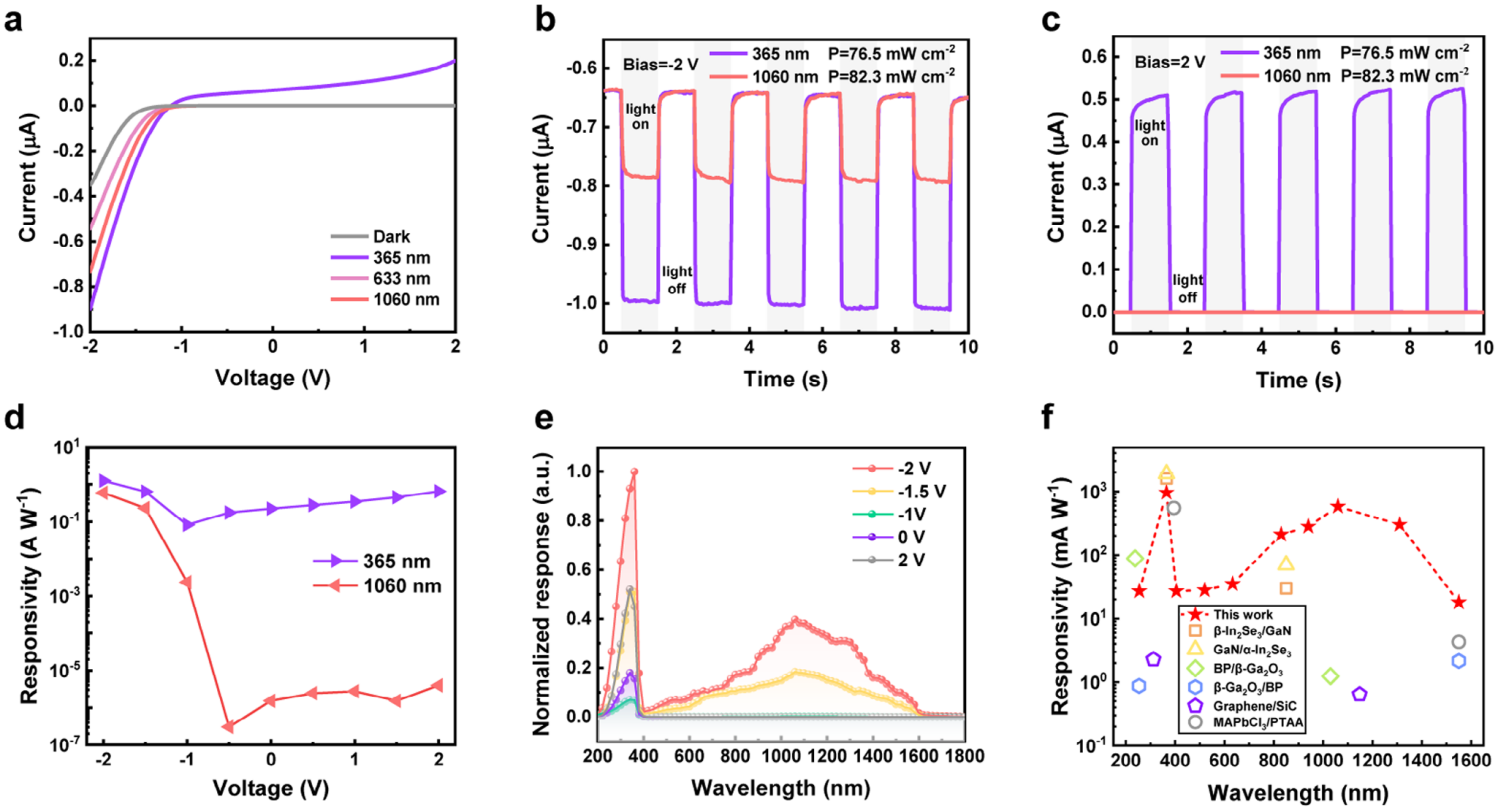
Bias‐tunable spectral response characterization of Te*_x_* Se_1‐_ *_x_*/GaN p‐P Heterojunction. a) *I‐*‐*V* curves of the bias‐tunable multispectral photodetector under dark conditions and light illumination at different wavelengths (365, 633, and 1060 nm). b,c) Time‐dependent current curves of the device at ±2 V bias when subjected to cyclic illuminations of 365 nm light and 1060 nm light. d) Responsivity as a function of bias voltages under light illuminations with 365 and 1060 nm wavelengths. e) The normalized spectral response of the device at different bias voltages. f) Comparison of responsivity of the device with previously reported UV‐IR dual‐band photodetectors.

Taking advantage of the feature of unidirectional barrier in the Te*_x_* Se_1‐_ *_x_*/GaN heterojunction, we elaborately demonstrated the bias‐tunable multispectral detection capability of our Te*_x_* Se_1‐_ *_x_*/GaN p‐P heterojunction photodetector. **Figure** **3**a illustrates the wavelength‐dependent *I*–*V* characteristics of the device. Under short‐wavelength illumination (365 nm), the device shows prominent photocurrents as the bias changes from negative to positive. Whereas photocurrents are observed only at relatively large negative voltages, under long‐wavelength illumination (633 and 1060 nm). The origin of photocurrent is further confirmed through photocurrent mapping, which indicates that the device exhibits a strong photoresponse mainly in the overlapping region between Te*_x_* Se_1‐_ *_x_* and GaN (Figure S14, Supporting Information). The time‐dependent current curves of the device at −2 and +2 V bias when subjected to cyclic illuminations of 365 and 1060 nm light also verify the bias‐tunable photoresponse (Figure 3b,c). At −2 V bias, the currents vary steadily and rapidly with the modulated light signals of both wavelengths. In contrast, at +2 V bias, the current amplitudes are not observable under 1060 nm light illumination but obviously under 365 nm light illumination. These results are consistent with the proposed working principle. Figure 3d shows the *R* of the device under 365 and 1060 nm irradiation as a function of the bias voltage. It can be seen that *R*_365_ is maintained at a relatively high level over the entire voltage range and increases with the absolute value of bias voltage. Note that the *R*_365_ reaches a minimum at −1 V bias, which indicates the presence of a large built‐in electric field in the junction region. For comparison, *R*_1060_ falls sharply when the voltage approaches −1.5 V and reaches a minimum at −0.5 V bias. Furthermore, *R*_1060_ does not increase with voltage under positive bias. Thus, spectral rejection ratios (*R*_365_/*R*_1060_) of ≈2.1 and ≈10^6^ are available at −2 and +2 V bias, respectively, suggesting that the single‐junction GaN/Te*_x_* Se_1‐_ *_x_* can extend the spectral response range from UV to IR by merely adjusting the bias voltage. For a clearer representation of the bias‐tunable multispectral response characteristic, we conducted the normalized spectral response measurements (Figure 3e). The spectral response of the device gradually switches from UV–Vis–IR to UV as the bias voltage changes from −2 to +2 V. At −2 and −1.5 V bias, two response peaks ≈360 and 1060 nm are observed, which correspond to the absorption cutoff edges of Te*_x_* Se_1‐_ *_x_* and GaN, respectively (Figure S2, Supporting Information). To characterize the broadband detection performance, we measured the photoresponse of the device to several typical wavelengths from UV to IR region at −2 V bias (Figures S15–S23, Supporting Information). The calculated *R* of different wavelengths is summarized in Figure 3f, which is comparable with that of previously reported UV‐IR dual‐band photodetectors. A more detailed comparison of UV and IR detection performance parameters is provided in Table S1 (Supporting Information). Notably, our Te_x_Se_1‐x_/GaN heterojunction demonstrates a competitive overall performance.

**Figure 4:**
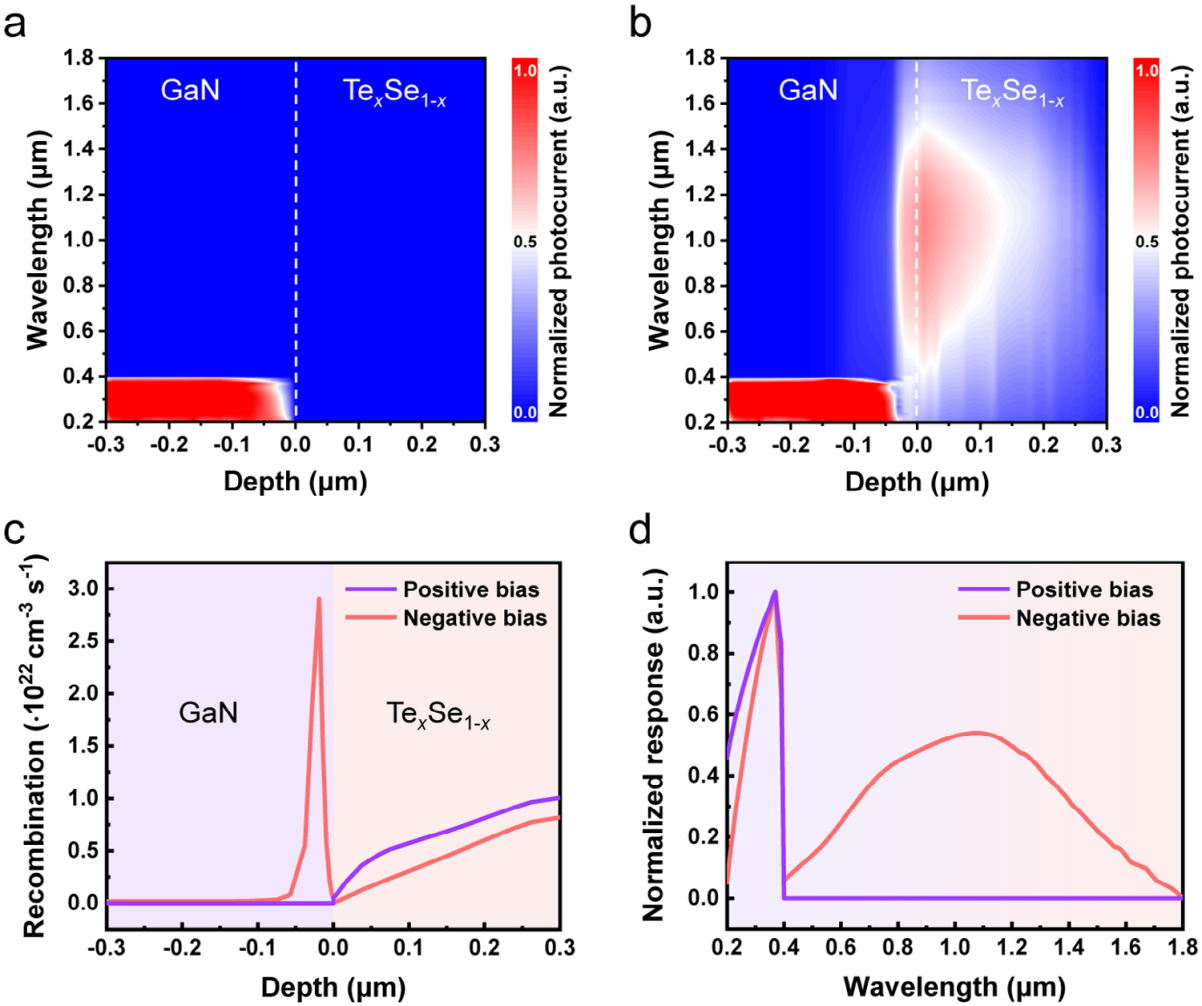
TCAD‐simulated bias‐tunable multispectral photodetection. a,b) TCAD‐simulated cross‐sectional photocurrent distribution of Te*_x_* Se_1‐_ *_x_*/GaN p‐P heterojunction working under positive bias and negative bias, respectively. c) TCAD‐simulated carrier recombination rate distribution across Te*_x_* Se_1‐_ *_x_*/GaN p‐P heterojunction under positive bias and negative bias. d) TCAD‐simulated photoresponse spectra of Te*_x_* Se_1‐_ *_x_*/GaN p‐P heterojunction under positive bias and negative bias.

To further understand the working principle of our bias‐tunable multispectral photodetector, Sentaurus TCAD is used here to visualize the photocurrent distribution and carrier transport in the Te*_x_* Se_1‐_ *_x_*/GaN p‐P heterojunction. From the cross‐section photocurrent distribution under positive bias in **Figure** **4**a, it can be seen that the photogenerated carriers excited in Te*_x_* Se_1‐_ *_x_* do not contribute to the photocurrent. When the UV light source is positioned near the heterointerface (white dash line in Figure 4a), the intensity of the photocurrent decreases significantly and ultimately reaches zero when the UV light is entirely directed at the Te*_x_* Se_1−_ *_x_* side, which determines that the photocurrent originates exclusively from GaN. In contrast, under negative bias, the photocurrent generated by visible and infrared irradiation is attributed to the absorption of Te*_x_* Se_1‐_ *_x_* (Figure 4b). This result is exactly corroborated by the recombination rate distribution as presented in Figure 4c. Under positive bias, the transport of the photogenerated holes in Te*_x_* Se_1‐_ *_x_* is highly inhibited by the valence band barrier, which brings about a high recombination rate on the Te*_x_* Se_1‐_ *_x_* side and no photocurrent generation. Whereas when a negative bias is applied, the photogenerated electrons in Te*_x_* Se_1‐_ *_x_* drift toward GaN and recombine with a large number of holes in the depletion region of GaN, resulting in the formation of a recombination current. Additionally, the good agreement between the simulated photoresponse spectra (Figure 4d) and the experimental results (Figure 3f) further validate the carrier transport process we proposed. More detailed TCAD simulation analyses of Te*_x_* Se_1‐_ *_x_*/GaN p‐P heterojunction are available in Figure S24 (Supporting Information). The above simulation results show that due to the presence of the unidirectional interfacial potential barrier, the bias voltage can significantly affect the collection efficiency of the photogenerated carriers and thus the response spectral range of the device.

### 2.4 Potential Applications Enabled by Bias‐Tunable Multispectral Detection

**Figure 5:**
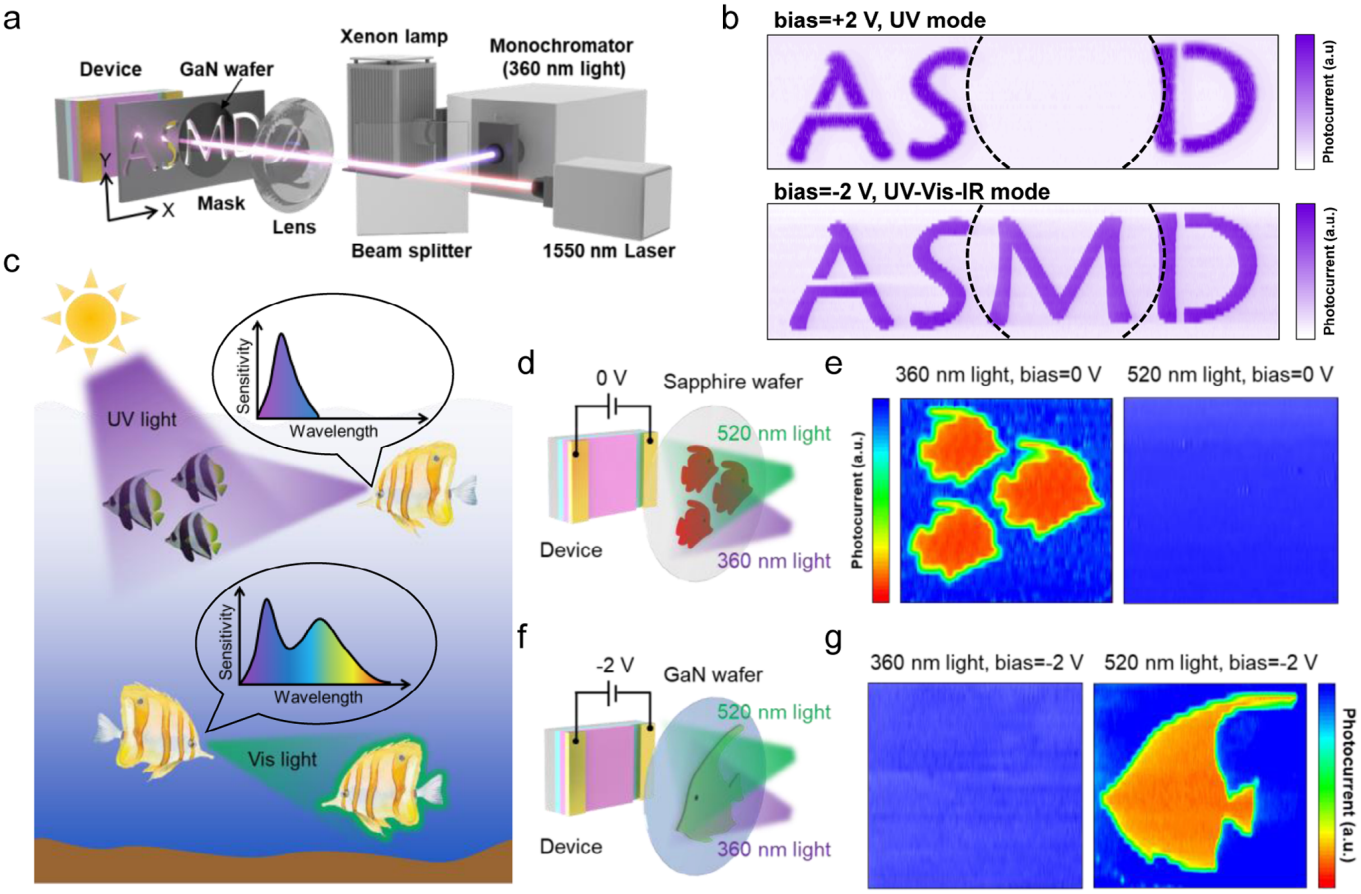
Potential applications enabled by bias‐tunable multispectral photodetection. a) The experimental setup diagram of the scanning imaging system. b) Scanning images of 360 nm UV light for the device working under ±2 V bias. c) Adjustable sensing spectral range of polyipnus spinous. d) Schematic of the visual effect simulation of polyipnus spinosus in shallow ocean environment. e) Scanning images with UV and Vis light for the device working under 0 V bias. f) Schematic of the visual effect simulation of polyipnus spinosus in the deep ocean environment. g) Scanning images with UV and Vis light for the device working under −2 V bias.

Finally, to demonstrate the potential application value of the designed bias‐tunable multispectral photodetector, two sets of proof‐of‐concept imaging were presented. We first verified the target recognition capability of the device in complex application scenarios. The schematic diagram of the test setup is presented in **Figure** **5**a. A metal mask with engraved letters “ASMD” is used as the imaging target and mounted on an xy‐axis displacement stage. In addition, the letter “M” is obscured by a GaN wafer. The 360 nm monochromatic light separated from a xenon lamp and 1550 nm laser are combined and simultaneously projected onto the metal mask. Figure 5b shows the scanning images by the device in UV mode (+2 V bias) and UV–Vis–IR mode (−2 V bias). Due to the UV photons being filtered out by the GaN wafer, the scanning image clearly displays the letters “A,” “S” and “D,” but fails to display the letter “M” in UV mode. In contrast, in UV–Vis–IR mode, the device is capable of sensing NIR photons transmitted through the GaN wafer, thus allowing the missing letter “M” to be displayed again. Recently, the development of artificial retinas that mimic the spectral adaptability of biological vision systems has been a growing area of interest.^[^ ^52^, ^53^, ^54^, ^55^ ^]^ Our device's ability to switch between UV and IR detection modes aligns with this trend, offering a simplified and cost‐effective solution for adaptive multispectral imaging. Motivated by the recent advancements in bioinspired artificial eye research, we successfully mimicked the visual systems of certain marine organisms, such as lanternfishes, which can adapt their spectral sensitivity to varying lighting conditions in the ocean.^[^ ^56^, ^57^ ^]^ As shown in Figure 5c, due to the weaker absorption of UV light by seawater, lanternfishes can utilize UV light for predation in the shallow sea. In the dim environment of the deep sea, however, lanternfishes may rely on their bioluminescent organs for illumination and communication, while simultaneously expanding the spectral range of their visual system to include visible waveband.^[^ ^58^, ^59^ ^]^ To simulate the visual effects of lanternfishes in both shallow and deep sea environments, the fish‐shaped patterns on sapphire and GaN wafers were used as imaging targets, respectively (Figure 5d‐f). At 0 V bias, the device can capture a clear image only under 360 nm UV radiation (Figure 5e). At −2 V bias, the visible light imaging was realized as expected (Figure 5g). This result demonstrates that our bias‐tunable multispectral photodetector can perform the functions of complex visual sensors and holds promise for applications in deep‐sea detection technology.

Compared to traditional multispectral detection technology our devices bring the following advantages to multispectral imaging: I) The bias‐dependent spectral response characteristic eliminates the dependence on mechanical filters and complex spectroscopic systems, simplifying the optical design; II) Bias adjustment and spectral switching are virtually delay‐free, suitable for high‐speed dynamic scene capture; III) Utilizing a single device to realize multispectral detection significantly reduces device size and production cost and improves system integrated level. In real‐world applications, it is necessary to monolithically integrate the array device with the readout integrated circuits (ROIC) and change the operating waveband of the imaging chip by switching the pixel bias to realize real‐time multispectral imaging. However, the implementation of bias polarity management via conventional ROIC technology is still a critical challenge at present. In the future, with the advancement of ROIC manufacturing processes, bias polarity switching may gradually become possible, and bias‐tunable multispectral imaging chips are expected to be achieved.

## 3 Conclusion

In summary, by the delicate design of band alignment, we fabricated a bias‐tunable multispectral photodetector utilizing a Te*_x_* Se_1‐_ *_x_*/GaN homo‐type heterojunction with a unidirectional barrier. In this structure, the majority‐carrier dark current is effectively suppressed by the unidirectional barrier, resulting in an extremely low reverse dark current of several pA and a high rectification ratio of ≈10^5^. By means of the unidirectional blockage effect of the interface barrier, the collection efficiency of photogenerated carriers in Te*_x_* Se_1‐_ *_x_* can be effectively regulated by merely altering the polarity and magnitude of the bias voltage. Thus, the spectral response range of the device is switched from UV waveband to UV–Vis–IR broadband when the applied bias changes from +2 to −2 V. At 0 V bias, the device achieves a responsivity of 0.25 A W^−1^ and a specific detectivity of 5.04 × 10^11^ cm Hz^1/2^ W^−1^ under 365 nm illumination. At −2 V bias, it exhibits a responsivity of 0.58 A W^−1^ and a specific detectivity of 2.64 × 10^9^ cm Hz^1/2^ W^−1^ under 1060 nm illumination. Furthermore, we demonstrated the proof‐of‐concept imaging enabled by bias‐tunable multispectral detection, indicating that the designed device embraces great potential for practical application. Our research provides a simple and cost‐effective approach for manufacturing multispectral photodetectors. By fully leveraging band alignment engineering and optimizing the device structure, it is believed that more advanced functionalities and superior performances can be realized in electrically tunable multispectral photodetectors.

## 4 Experimental Section

### Device Fabrication

The GaN epitaxial layer was etched into rectangular strips by inductively coupled plasma (ICP) etching. Te*_x_* Se_1‐_ *_x_* thin film was deposited to form Te*_x_* Se_1‐_ *_x_*/GaN heterojunction by thermal evaporation. Ni/Au (20/30 nm) and Au (50 nm) as contact electrodes for GaN and Te*_x_* Se_1‐_ *_x_*, respectively, were deposited using electron beam evaporation (EBE). The GaN etching region, Te*_x_* Se_1‐_ *_x_* deposition region, and metal electrodes deposition region were defined using maskless lithography. A detailed description of the device fabrication process is given in Figure S1 (Supporting Information).

### Material and Structure Characterizations

OM images were taken by an optical microscope (RX50 M, SOPTOP). The absorption spectra were measured using a UV–Vis–NIR Spectrometer (UV‐3600 Plus, Shimadzu) to obtain the bandgaps. Ultraviolet photoelectron spectroscopy (AXIS SUPRA+, Kratos) was utilized to determine the valence band and Fermi level. The heterojunction interface quality and the bond vibration modes were observed by transmission electron microscopy (Tecnai G2 F30, FEI) and Raman spectrometer (LabRAM HR800, Horiba Jobin Yvon), respectively.

### Electrical and Optoelectronic Measurements

The electrical and optoelectronic performances were measured using the Keysight B2902A source connected to a photocurrent probe station (NANOBASE, XperPC), under room temperature and ambient conditions. Visible and infrared light sources were provided by lasers equipped with pulse generators. The ultraviolet light source was provided by a 365 nm LED, which was driven and modulated by a function generator (DG5252, Rigol). The excitation light for spectral response measurement was provided by the combination of a white light source and a monochromator. The light intensity was calibrated with a commercial optical power meter (Thorlabs PM100D). High time‐resolution response current signal was converted to a voltage signal using a preamp (Stanford Research Systems SR570) and recorded by Tektronix MDO3014 mixed domain oscilloscope. The noise current spectral was gauged using a current preamplifier (Stanford Research Systems SR570) and a signal analyzer (Keysight EXAN9010B).

## Conflict of Interest

The authors declare no conflict of interest.

## Supporting information



Supporting Information

## Data Availability

The data that support the findings of this study are available from the corresponding author upon reasonable request.
