# Supplementary material for: A Bias‐Tunable Multispectral Photodetector Based on a GaN/Te x Se1‐ x Homo‐Type Heterojunction with a Unidirectional Barrier
Source: Adv Sci (Weinh). 2025 Mar 7;12(17):2417428. doi: 10.1002/advs.202417428 (PMC12061333; doi:10.1002/advs.202417428)
Supplement: Supplementary file 1 — Supporting Information [file ADVS-12-2417428-s001.docx]

Supporting Information

A Bias-Tunable Multispectral Photodetector Based on a GaN/Te*_x_*Se_1-_*_x_* Homo-type Heterojunction with a Unidirectional Barrier

Weijie Liu, Meng Peng, Maohua Chen, Yongming Zhao, Yiye Yu, Pengcheng Jian, Zunyu Liu, Yuhui Zeng, Yuang Luo, Xiantai Tian, Zhiwei Gao, Jiangnan Dai, Changqing chen, Feng Wu*, and Weida Hu*

W. Liu, M. Peng, M. Chen, Y. Zhao, Y. Yu, P. Jian, Z. Liu, Y. Zeng, X. Tian, Z. Gao, Prof. J. Dai, Prof. F. Wu

Wuhan National Laboratory for Optoelectronics, Huazhong University of Science and Technology, Wuhan 430074, China

E-mail: [wufeng123@hust.edu.cn](mailto:wufeng123@hust.edu.cn);

Y. Luo, Prof. C. Chen

School of Optical and Electronic Information, Huazhong University of Science and Technology, Wuhan 430074, China

Y. Yu, Prof. W. Hu

State Key Laboratory of Infrared Science and Technology, Shanghai Institute of Technical Physics, Chinese Academy of Sciences, Shanghai 200083, China

Email: [wdhu@mail.sitp.ac.cn](mailto:wdhu@mail.sitp.ac.cn);

**Figure S1.**  Schematic diagram of device fabrication process.

**Step 1. GaN growth.** The p-type doped GaN (Mg doping concentration of 4 × 10^17^ cm^-3^) was epitaxial grown on sapphire substrate with a 1 μm thick AlN buffer by metalorganic chemical vapor deposition (MOCVD). Then, the GaN epitaxial wafer was ultrasonically cleaned with dilute hydrochloric acid, acetone, isopropanol, and deionized water, each for 15 minutes, to clean off contaminants.

**Step 2. GaN etching.** The GaN epitaxial layer was etched into rectangular strips by inductively coupled plasma (ICP) etching at a Cl_2_/BCl_3_ hybrid atmosphere. The GaN etching region was defined using maskless lithography.

**Step 3. Ni/Au electrodes deposition.** The contact electrodes of GaN were defined by maskless lithography, followed by deposition of Ni/Au metal stack (20/30 nm) using electron beam evaporation (EBE) and standard lift-off process in acetone. To achieve ohmic contact between GaN and electrodes, the sample was annealed at 700 ℃ for 10 min in air atmosphere.

**Step 4. Te*_x_*Se_1-_*_x_* thin film deposition.** After the deposition region was defined by maskless lithography, 300 nm thick Te*_x_*Se_1-_*_x_* thin film was deposited to from Te*_x_*Se_1-_*_x_*/GaN heterojunction by thermal evaporation with the Se powder (99.99%, Sigma Aldrich) and Te powder (99.999%, Sigma Aldrich) as the source materials (at 1.5 × 10^−4^ Pa; Se ≈ 1.6 Å s^−1^, Te ≈ 2.4 Å s^−1^). Then, the sample was annealed on a hotplate at 240 °C for 5 min to improve the thin film quality of the Te*_x_*Se_1-_*_x_*. The annealing process was conducted in a nitrogen glove box to prevent oxidation of the Te*_x_*Se_1-_*_x_* thin film.

**Step 5. Au electrodes deposition.** Finally, 50 nm thick Au electrodes were deposited on Te*_x_*Se_1-_*_x_* thin film using EBE. The electrode region was defined using maskless lithography.

**Figure S2.** Bandgap measurements for Te*_x_*Se_1-_*_x_* and GaN. (a), (b) absorption spectrum and Tauc plot of Te*_x_*Se_1-_*_x_*. (c), (d) absorption spectrum and Tauc plot of GaN.

1. **Figure S3.**  UPS spectra of Te*_x_*Se_1-_*_x_* and GaN displaying the valence band spectra and second electron cutoffs. (a), (b) Valence band spectrum, and second electron cutoffs of Te*_x_*Se_1-_*_x_*. (c), (d) Valence band spectrum, and second electron cutoffs of GaN.

The UPS spectra are utilized to confirm the valence band and work function of Te*_x_*Se_1-_*_x_* and GaN. The work functions of Te*_x_*Se_1-_*_x_* and GaN were estimated to be 4.59 and 7.29 eV, according to W = hν - E_cut_, where hν = 21.22 eV is the photon energy of He I light source. Their Fermi levels are located 0.02 and 0.21 eV above the valence bands for Te*_x_*Se_1-_*_x_* and GaN, respectively.

**Figure S4.** Schematic band alignments of Te*_x_*Se_1-_*_x_* and GaN. (a) The band alignments before contact and (b) after contact (zore bias).

1. **Figure S5.** Raman spectra of Te*_x_*Se_1-_*_x_*, and GaN. (a) Raman spectrum of Te*_x_*Se_1-_*_x_*. (b) Raman spectrum of GaN. The wavelength of the excitation laser is 532 nm.

The typical Raman spectrum peaks of Te*_x_*Se_1-_*_x_* are located at 149 (Te-like A_1_), 176 (Te-like E_2_) and 216 (Se-like E_2_) cm^-1^. The typical Raman spectrum peaks of GaN are located at 574 (E_2_ high) and 748 (A_1_ LO) cm^-1^, respectively. Raman spectra of above all are consistent with the previous work ^[1-3]^.

**Figure S6.** XPS spectra of Te*_x_*Se_1-_*_x_* and GaN. (a) Te 3d XPS peaks, (b) Te 3d XPS peaks, (c) Ga 3p XPS peaks and (d) N 1s XPS peaks with fitted characteristic peaks.

**Figure S7.** X-ray diffraction (XRD) patterns of the Te_x_Se_1-x_ thin film.

**Figure S8.** Selected area electron diffraction (SAED) pattern of Te_x_Se_1-x_ thin film.

1. **Figure S9.** Exploration of p-GaN ohmic contact processes. (a) The optical image of circular transmission line model (CTLM) test pattern. (b) The current-voltage relation (*I*-*V*) curves of p-GaN after thermal annealing at different temperatures. (c) *I*-*V* curves of p-GaN (annealing at 700 ℃) for different electrode spacing (*d*). (d) Total resistance (*R_T_*) versus *d* and the corresponding linear fitting curve.

**Figure S10.**  Current-voltage relation (*I*-*V*) curves of Te*_x_*Se_1-_*_x_* and GaN. (a) *I*-*V* curve of Te*_x_*Se_1-_*_x_*. (b) *I-V* curve of GaN.

**Figure S11.** Current-voltage relation (*I*-*V*) curves of Te*_x_*Se_1-_*_x_*/GaN p-N heterojunction.

**Figure S12.** Schematic band alignment of Te*_x_*Se_1-_*_x_*/GaN p-N heterojunction under negative bias.

1. **Figure S13.** Noise characteristics for the Te*_x_*Se_1-_*_x_*/GaN p-P heterojunction under different bias voltage. (a) Spectral noise density at -2 V bias. (b) Spectral noise density at 0 V bias. (c) Spectral noise density at 2 V bias.

At -2 V bias, the noise power is inversely proportional to the frequency, indicating that the 1/*f* noise is dominant noise type. While, at 0 and 2 V bias, the noise power is almost independent of frequency, suggesting that thermal and shot noise are dominant noise types.

**Figure S14.** Photocurrent mappings of Te*_x_*Se_1-_*_x_*/GaN p-P heterojunction under various wavelength lasers. (a) 633 nm. (b) 1060 nm.

1. **Figure S15.**  Optoelectronic Performance of Te*_x_*Se_1-_*_x_*/GaN p-P Heterojunction for 365 nm wavelength. (a) Time-dependent photoresponse of the Te*_x_*Se_1-_*_x_*/GaN p-P heterojunction at -2 V bias with different power density of 365 nm wavelength. (b) *R* and *D*^*^ versus power densities of 365 nm wavelength.
2.
3. **Figure S16.** Optoelectronic Performance of Te*_x_*Se_1-_*_x_*/GaN p-P Heterojunction for 405 nm wavelength. (a) Time-dependent photoresponse of the Te*_x_*Se_1-_*_x_*/GaN p-P heterojunction at -2 V bias with different power density of 405 nm laser. (b) *R* and *D*^*^ versus power densities of 405 nm laser.
4.
5. **Figure S17.**  Optoelectronic Performance of Te*_x_*Se_1-_*_x_*/GaN p-P Heterojunction for 520 nm wavelength. (a) Time-dependent photoresponse of the Te*_x_*Se_1-_*_x_*/GaN p-P heterojunction at -2 V bias with different power density of 520 nm laser. (b) *R* and *D*^*^ versus power densities of 520 nm laser.

**Figure S18.**  Optoelectronic Performance of Te*_x_*Se_1-_*_x_*/GaN p-P Heterojunction for 633 nm wavelength. (a) Time-dependent photoresponse of the Te*_x_*Se_1-_*_x_*/GaN p-P heterojunction at -2 V bias with different power density of 633 nm laser. (b) *R* and *D*^*^ versus power densities of 633 nm laser.

1. **Figure S19.** Optoelectronic Performance of Te*_x_*Se_1-_*_x_*/GaN p-P Heterojunction for 830 nm wavelength. (a) Time-dependent photoresponse of the Te*_x_*Se_1-_*_x_*/GaN p-P heterojunction at -2 V bias with different power density of 830 nm laser. (b) *R* and *D*^*^ versus power densities of 830 nm laser.

**Figure S20.** Optoelectronic Performance of Te*_x_*Se_1-_*_x_*/GaN p-P Heterojunction for 940 nm wavelength. (a) Time-dependent photoresponse of the Te*_x_*Se_1-_*_x_*/GaN p-P heterojunction at -2 V bias with different power density of 940 nm laser. (b) *R* and *D*^*^ versus power densities of 940 nm laser.

**Figure S21.** Optoelectronic Performance of GaN/Te*_x_*Se_1-_*_x_* p-P Heterojunction for 1060 nm wavelength. (a) Time-dependent photoresponse of the Te*_x_*Se_1-_*_x_*/GaN p-P heterojunction at -2 V bias with different power density of 1060 nm laser. (b) *R* and *D*^*^ versus power densities of 1060 nm laser.

**Figure S22.** Optoelectronic Performance of Te*_x_*Se_1-_*_x_*/GaN p-P Heterojunction for 1310 nm wavelength. (a) Time-dependent photoresponse of the Te*_x_*Se_1-_*_x_*/GaN p-P heterojunction at -2 V bias with different power density of 1310 nm laser. (b) *R* and *D*^*^ versus power densities of 1310 nm laser.

1. **Fig. S23.** Optoelectronic Performance of Te*_x_*Se_1-_*_x_*/GaN p-P Heterojunction for 1550 nm wavelength. (a) Time-dependent photoresponse of the Te*_x_*Se_1-_*_x_*/GaN p-P heterojunction at -2 V bias with different power density of 1550 nm laser. (b) *R* and *D*^*^ versus power densities of 1550 nm laser.

**Figure S24.**  TCAD device simulation of Te*_x_*Se_1-_*_x_*/GaN p-P Heterojunction. (a) Current density (vector). (b) Optical generation. (c) Hole density. (d) Recombination contours of Te*_x_*Se_1-_*_x_*/GaN p-P Heterojunction at positive bias (left panel) and negative bias (right panel), respectively.

When positive bias is applied, the current flows from Te*_x_*Se_1-_*_x_* to GaN. And the current flows in the opposite direction under negative bias. However, the optical generation does not vary with bias polarity. Under positive bias, the photogenerated holes in Te*_x_*Se_1-_*_x_* are blocked by the unidirectional barrier at the heterojunction interface, and thus there is a large accumulation of holes on Te*_x_*Se_1-_*_x_* side, leading to a high recombination rate and no effective photocurrent generation. Under negative bias, the carrier transport direction is reversed, and the photogenerated electron-hole pairs generated in Te*_x_*Se_1-_*_x_* can be smoothly collected by the electrodes, and thus photocurrents are formed. In other words, while the bias voltage has little impact on light-matter interactions, it significantly affects carrier transport processes. This explains why the spectral response of the device changes with variations in the bias voltage.

| 1. **Device structure** | 1. **Responsivity @ λ** | 1. **Detectivity** ( **cm Hz^1/2^ W^-1^ ) @ λ** | 1. **Rise/fall time @ λ** | 1. **Ref.** |
| --- | --- | --- | --- | --- |
| 1. GaN/AlGaN | 1. 3.8 mA/W @360 nm 2. 85 mA/W @5 μm | 1. - | 1. - | 1. [4] |
| 1. β-Ga_2_O_3_/BP | 1. 88.5 mA/W @238 nm 2. 1.24 mA/W @1030 nm | 1. - | 1. 2.1/6.1 s @250 nm 2. 0.25/0.31 s @1025 nm | 1. [5] |
| 1. β-In_2_Se_3_/GaN | 1. 1.6 A/W @365 nm 2. 0.03 A/W @850 nm | 1. 3.3×10^11^ @ 365 nm 2. 1.6×10^9^ @850 nm | 1. 0.22/0.21 s @365 nm 2. 12/6.4 s @850 nm | 1. [6] |
| 1. Graphene/SiC | 1. 2.27 mA/W @312 nm 2. 0.64 mA/W @1148 nm | 1. - | 1. - | 1. [7] |
| 1. GaN nanowire/Graphene | 1. 6.93 A/W @360 nm 2. 0.11 A/W @1540 nm | 1. 1.23×10^12^ @ 360 nm 2. 1.88×10^10^ @1540 nm | 1. 0.36/1.22 ms @360 nm 2. 0.76/2.68 ms @1540 nm | 1. [8] |
| 1. Cs_3_Cu_2_I_5_/PdTe_2_/Ge | 1. 744.2 mA/W @265 nm 2. 712.5 mA/W @1550 nm | 1. 5.08×10^9^ @ 265 nm 2. 1.37×10^11^ @1550 nm | 1. 3.8/3.1 s @265 nm 2. 24.9/26.2 μs @1550 nm | [9] |
| 1. p-GaN/α-In_2_Se_3_ | 1. 1.9 A/W @365 nm 2. 0.07 A/W @850 nm | 1. 4.6×10^10^ @ 365 nm 2. 3.6×10^10^ @ 850 nm | 1. 0.26/0.13 s @365 nm 2. 0.2/0.42 s @850 nm | [10] |
| β-Ga_2_O_3_/BP | 1. 2.39×10^6^ mA/W @254 nm 2. 535 mA/W @808 nm | 1. 2.4×10^10^ @ 254 nm 2. 6.1×10^6^ @ 808 nm | 1. 0.5/5.9 s @254 nm 2. 0.6/11 s @808 nm | [11] |
| 1. β-Ga_2_O_3_/Te | 1. 3.82×10^6^ A/W @255 nm 2. 138 A/W @1550 nm | 1. 1.59×10^14^ @ 255 nm 2. 3.7×10^9^ @ 1550 nm | 1. 92/95 ms @255 nm 2. 43.4/38.1 ms @1550 nm | [12] |
| 1. MAPbCl_3_/PTAA | 1. 550 mA/W @395 nm 2. 4.3 mA/W @1550 nm | 1. 2.21×10^11^ @ 395 nm 2. 1.7×10^9^ @ 1550 nm | 1. 11.1/36.1 μs @390 nm | [13] |
| 1. Te*_x_*Se_1-_*_x_*/p-GaN | 1. 0.96 A/W @365 nm 2. 0.58 A/W @1060 nm | 1. 5.04×10^11^ @ 365 nm 2. 2.64×10^9^ @ 1060 nm | 1. 0.51/0.64 ms @365 nm 2. 0.13/0.16 ms @1060 nm | 1. This work |

1. **Table S1.** Ultraviolet and infrared detection performance parameters of the Te*_x_*Se_1-_*_x_*/GaN bias-tunable multispectral photodetector in comparison with the previously reported ultraviolet-infrared dual-band photodetectors.

**Note S1.** Calculation of specific contact resistivity of p-GaN

In order to obtain low-resistance ohmic contacts of p-GaN, we conducted rapid thermal annealing (RTP) at different temperatures. From the Fig. S7b, it can be seen that after annealing at 700°C, the current increases significantly and the current-voltage relation (*I*-*V*) curve shows a good linear relationship. The specific contact resistivity (*ρ*_c_) of p-GaN was extracted by circular transmission line model (CTLM)^[14]^. The CTLM method requires the fabrication of a set of circular electrodes on the surface of the material, each with the same radius (*r*_0_) of 100 µm as shown in Fig. S7a. Additionally, a ring-shaped blank area was left outside each circular metal electrode, with the outer diameters (*r_n_*) of the rings being 120, 130, 140, 150, 160, and 170 µm, respectively. The total resistance (*R*_T_) between the electrodes on either side of the ring can be expressed as:

(Equation S1)

where *I* and *K* are the modified Bessel functions of the first and second kind respectively, *R_sh_* is the sheet resistance of p-GaN and *L_T_* is the transfer length given by:

$L_{T}=\sqrt{\frac{\rho_{c}}{R_{sh}}}$ (Equation S2)

When *r*_0_ and *r_n_* are much greater than *L_T_*, both *I*_0_/*I*_1_ and *K*_0_/*K*_1_ approximate to unity. Thus, Equation (S1) becomes:

$R_{T}=\frac{R_{sh}}{2\pi}\left[\ln\left(\frac{r_{n}}{r_{n}-d}\right)+L_{T}\left(\frac{1}{r_{n}-d}+\frac{1}{r_{n}}\right)\right]$ (Equation S3)

Where *d* = *r_n_ - r*_0_. If *r*_n_ >> *d*, the above reduces to:

$R_{T}=\frac{R_{sh}}{2\pi r_{0}}\left(d+2L_{T}\right)$ (Equation S4)

By measuring the *I*-*V* curves between the electrodes on either side of each ring (Fig. S7c), *R_T_* can be determined. Subsequently, the *R_T_* - d curve was linearly fitted according to Equation S4, from which *R_sh_ =* 162.88 KΩ and *L_T_ =* 1.53 μm were obtained from the slope and intercept of the resulting line, respectively (Fig. S7d). The calculated *R_sh_* and *L_T_* were then substituted into Equation S2 to determine *ρ*_c_ = 3.81×10^-3^ Ω cm^2^, which is in close agreement with previously reported values, thereby demonstrating the successful achievement of low-resistance Ohmic contact of p-GaN.

**References**

[1] H. Huang, J. Zha, S. Xu, P. Yang, Y. Xia, H. Wang, D. Dong, L. Zheng, Y. Yao, Y. Zhang, Y. Chen, J. C. Ho, H. P. Chan, C. Zhao, C. Tan, *ACS Nano* **2024**, 18, 17293.

[2] C. Tan, M. Amani, C. Zhao, M. Hettick, X. Song, D. H. Lien, H. Li, M. Yeh, V. R. Shrestha, K. B. Crozier, M. C. Scott, A. Javey, *Adv. Mater.* **2020**, 32, 2001329.

[3] D. K. Singh, R. K. Pant, K. K. Nanda, S. B. Krupanidhi, *Appl. Phys. Lett.* **2021**, 119, 121102.

[4] A. G. U. Perera, G. Ariyawansa, M. B. M. Rinzan, M. Stevens, M. Alevli, N. Dietz, S. G. Matsik, A. Asghar, I. T. Ferguson, H. Luo, A. Bezinger, H. C. Liu, *Infrared Physics & Technology* **2007**, 50, 142.

[5] T. He, C. Li, X. Zhang, Y. Ma, X. Cao, X. Shi, C. Sun, J. Li, L. Song, C. Zeng, K. Zhang, X. Zhang, B. Zhang, *Phys. Status Solidi A-Appl. Mat.* **2019**, 217, 1900861.

[6] S. V. Solanke, S. Rathkanthiwar, A. Kalra, R. K. Mech, M. Rangarajan, S. Raghavan, D. N. Nath, *Semicond. Sci. Technol.* **2019**, 34, 075020.

[7] C.-h. Zeng, W.-k. Lin, Y.-h. Sun, Q. Cui, X. Zhang, S.-j. Li, B.-s. Zhang, M. Kong, *Optoelectron. Lett.* **2019**, 15, 170.

[8] C. Zeng, W. Lin, T. He, Y. Zhao, Y. Sun, Q. Cui, X. Zhang, S. Lu, X. Zhang, Y. Xu, M. Kong, B. Zhang, *Chin. Opt. Lett.* **2020**, 18, 112501.

[9] Y. Liang, C. Xie, C.-y. Dong, X.-w. Tong, W.-h. Yang, C.-y. Wu, L.-b. Luo, *Journal of Materials Chemistry C* **2021**, 9, 14897.

[10] S. V. Solanke, R. Soman, M. Rangarajan, S. Raghavan, D. N. Nath, *Sens. Actuators A-Phys.* **2021**, 317, 112455.

[11] T. Chen, J. Zhang, X. Zhang, C. Chen, L. Zhang, Y. Hu, Y. Ma, X. Wei, X. Zhou, W. Tang, A. Yang, B. Li, S. Dai, L. Xu, W. Shi, H. Fu, Y. Fan, Y. Cai, Z. Zeng, K. Zhang, B. Zhang, *IEEE Sens. J.* **2023**, 23, 15504.

[12] W. Liu, M. Peng, Y. Yu, Z. Zheng, P. Jian, Y. Zhao, Y. Zeng, D. Xu, M. Chen, Y. Luo, C. Chen, J. Dai, F. Wu, *Adv. Opt. Mater.* **2024**, 12, 2302746.

[13] S. Liu, Y. Ding, W. Rong, Y. Xu, Y. Li, D. C. Onwudiwe, B. S. Bae, M. Ertuğrul, Y. Zhu, Z. Wu, W. Lei, Q. Li, X. Xu, *ACS Nano* **2024**, 18, 25226.

[14] G. S. Marlow, M. B. Das, *Solid-State Electron.* **1982**, 25, 91.
